# LASSO-derived prognostic model predicts cancer-specific survival in advanced pancreatic ductal adenocarcinoma over 50 years of age: a retrospective study of SEER database research

**DOI:** 10.3389/fonc.2023.1336251

**Published:** 2024-01-15

**Authors:** Yuan Feng, Junjun Yang, Wentao Duan, Yu Cai, Xiaohong Liu, Yong Peng

**Affiliations:** Department of Hepatobiliary Pancreatic and Spleen Surgery, Nanchong Central Hospital, The Second Clinical Medical College, North Sichuan Medical College, Nanchong, China

**Keywords:** advanced pancreatic ductal adenocarcinoma, nomogram, AJCC staging, risk stratification, cancer-specific survival

## Abstract

**Background:**

This study aimed to develop a prognostic model for patients with advanced ductal adenocarcinoma aged ≥50 years.

**Methods:**

Patient information was extracted from the Surveillance, Epidemiology, and End Results (SEER) database. Least absolute shrinkage and selection operator (LASSO) Cox regression analysis was performed to screen the model variables. Cases from Nanchang Central Hospital were collected for external validation. The new nomogram and the American Joint Committee on Cancer (AJCC) criteria were evaluated using integrated discrimination improvement (IDI) and net reclassification index (NRI) indicators. Survival curves presented the prognosis of the new classification system and AJCC criteria.

**Results:**

In total, 17,621 eligible patients were included. Lasso Cox regression selected 4 variables including age, chemotherapy, radiotherapy and AJCC stage. The C-index of the training cohort was 0.721. The C-index value of the validation cohort was 0.729. The AUCs for the training cohorts at 1, 2, and 3 years were 0.749, 0.729, and 0.715, respectively. The calibration curves showed that the predicted and actual probabilities at 1, 2, and 3 years matched. External validation confirmed the model’s outstanding predictive power. Decision curve analysis indicated that the clinical benefit of the nomogram was higher than that of the AJCC staging system. The model evaluation indices preceded the AJCC staging with NRI (1-year: 0.88, 2-year: 0.94, 3-year: 0.72) and IDI (1-year: 0.24, 2-year: 0.23, 3-year: 0.22). The Kaplan–Meier curves implied that the new classification system was more capable of distinguishing between patients at different risks.

**Conclusions:**

This study established a prognostic nomogram and risk classification system for advanced pancreatic cancer in patients aged ≥50 years to provide a practical tool for the clinical management of patients with pancreatic ductal adenocarcinoma.

## Background

Ductal adenocarcinoma of the pancreas is a fatal malignancy with the lowest five-year survival rate of all malignancies (1, 2). In the last decade, the mortality rate of pancreatic ductal adenocarcinoma has increased annually (3). The lack of obvious symptoms and lack of specific diagnostic techniques in the early stages of ductal adenocarcinoma of the pancreas has resulted in most patients not being detected until the advanced stages. Surgery is an effective treatment modality for pancreatic ductal adenocarcinoma; however, patients with advanced disease are deprived of surgical treatment (4–6). Induction therapy, chemotherapy, radiotherapy, and immunotherapy are the main modalities of treatment for advanced pancreatic ductal adenocarcinoma (7, 8).

Age is an influential factor in the incidence and mortality of pancreatic cancer. Recent studies have demonstrated that the incidence of pancreatic cancer is increasing every year worldwide. A population-based study found that only 10% of 10,298 patients included were younger than 50 years of age (9–11). Therefore, an age limit of >50 years was intended to identify our study population more accurately. AJCC staging is a common tool in the management of patients with pancreatic cancer. However, AJCC staging only considers neoplasm size and infiltration, and important factors affecting the prognosis of pancreatic ductal adenocarcinoma such as age and CA19-9, were not included (12). Ductal adenocarcinoma of the pancreas is a highly heterogeneous neoplasm, and survival prognosis varies widely among patients (13, 14). Therefore, there is a need to develop a personalized predictive tool to assist in the clinical management of patients with pancreatic ductal adenocarcinoma.

The nomogram has the advantage of being a visual tool and incorporating more clinical characteristics and are widely applied in oncology (15–17). In this study, information on advanced pancreatic ductal adenocarcinoma in patients aged ≥50 years was obtained from the SEER database. LASSO-based regression was performed to screen model variables and to develop a nomogram and risk classification system for patients with advanced pancreatic ductal adenocarcinoma aged ≥50 years.

## Methods

### Patient population and study variables

**Figure 1:**
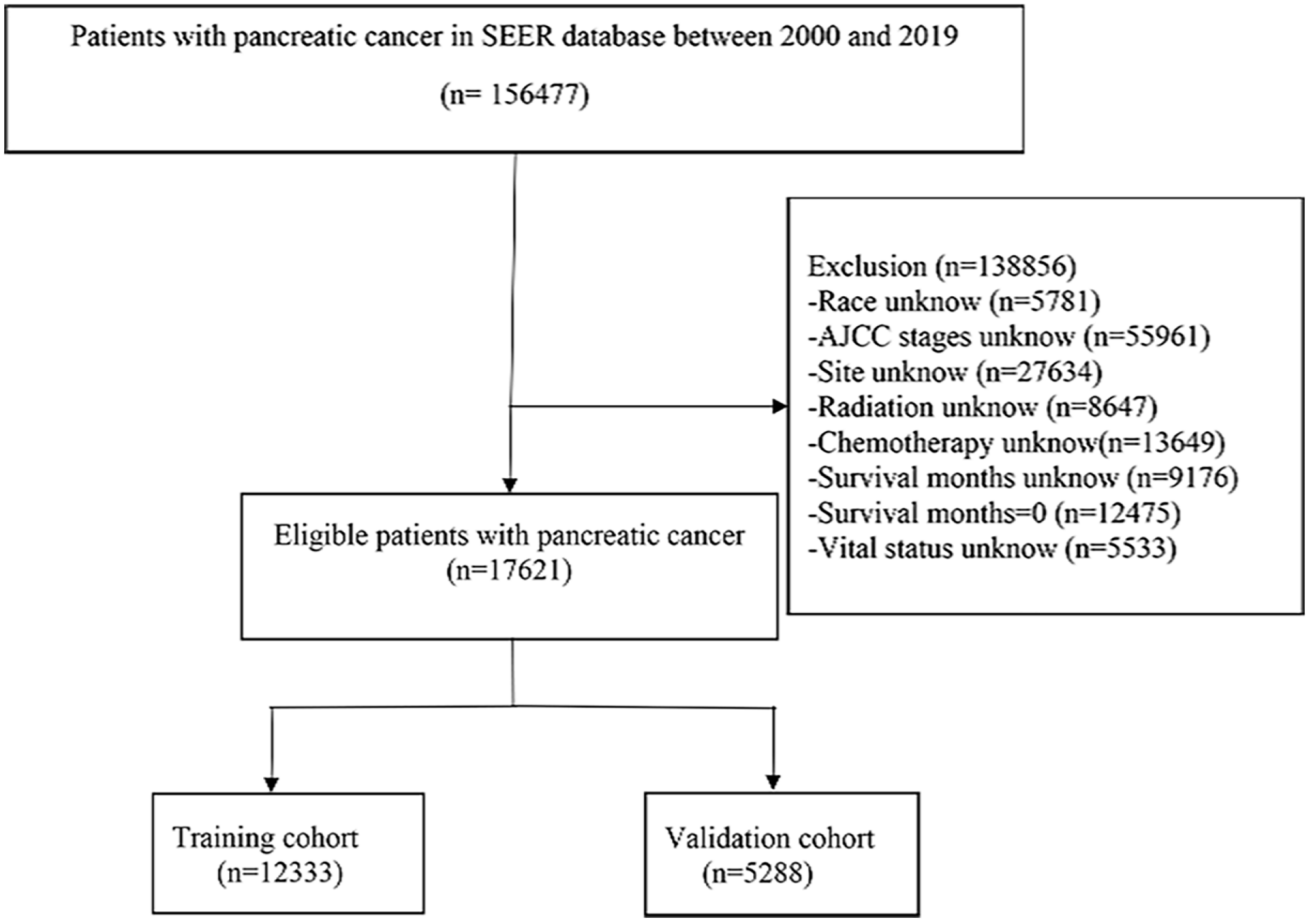
Screening process of the patients with advanced pancreatic ductal adenocarcinoma aged ≥50 years.

Patient information was downloaded from the SEER database, which contains basic and treatment information for most oncology patients. The inclusion criteria were as follows: (a) pathological type of adenocarcinoma of the pancreatic duct, (b) detailed treatment information, (c) clear cause of death, and (d) age ≥50 years. Exclusion criteria were as follows (a) primary tumor not pancreatic, (b) incomplete treatment information, (c) unknown cause of death, (d) survival time of 0, and (e) unknown AJCC stage (**Figure 1**). C25.0–25.9 of the International Classification of Diseases for Oncology, 3rd Revision (ICD-O-3), was used to determine the site of pancreatic ductal adenocarcinoma. By examining the clinical data of patients recorded in the SEER database and referring to risk factors for pancreatic cancer patients in previous studies, age, sex, CA19-9, race, grade, site, number, AJCC stage, chemotherapy, and radiotherapy were selected as the appropriate variables to be investigated. The endpoint of the study is cancer-specific survival (CSS), which is the time between the diagnosis of pancreatic cancer and death due to pancreatic cancer.

### Building the model

The least absolute shrinkage and selection operator (LASSO) Cox regression was applied to screen for model variables in advanced pancreatic ductal adenocarcinoma. Various methods have been employed to assess the predictive accuracy of the model, such as the C-index, receiver operating characteristic curves, and calibration curves. Decision curves were used to measure the clinical benefits of the nomograms.

### External validation

A total of 149 patients with advanced pancreatic cancer aged ≥50 years were recruited from Nanchang Central Hospital. Written informed consent was obtained from all the patients participating in the study. The stability of the model was verified by calculating the C-index and ROC and calibration curves.

### Comparison of the new model with the old model

The net reclassification index (NRI), integrated discrimination improvement (IDI), consistency index (C-index), and decision curve analysis (DCA) were used to estimate the practical applicability of the new model. The NRI and IDI indices were deployed to estimate the improved level of the new model compared with the AJCC. The C-index clearly demonstrated the high and low predictive power of the new model and AJCC staging.

### The new classification system

Based on the scoring system of the nomogram, the total risk score of all patients was calculated. Based on the total score, all patients were divided into low-, middle-, and high-risk groups (X-tile software was applied to select the best cut-off value between groups).

### Prognosis comparison

The AJCC staging system is the most accepted clinical tool for prognostic evaluation. Differences in the accuracy of the new risk classification system and AJCC staging in determining patient prognosis were compared by Kaplan–Meier curves.

### Data analysis

Patient information was extracted using SEER*Stat software (https://seer.cancer.gov/seerstat/). All data analyses were performed using R software (version 3.6.1; http://www.r-project.org/) and related packages. The cut-off values for the risk classification were obtained with the X-tile software (version 3.6.1). A chi-square test was applied to compare the distribution of data between the training and validation groups for statistical differences. P-values were all two-sided statistical tests, and P-values less than 0.05 were considered statistically significant. This work was in line with the STROCSS criteria (18).

## Results

### Patient characteristics

A total of 17,621 screened and eligible cases of pancreatic ductal adenocarcinoma aged ≥50 years were included in the study. A 7:3 ratio of random allocation resulted in the training (12,333 patients) and validation cohorts (5,288 patients). Approximately 48.72% of patients were aged between 65 and 80 years. The percentage of patients who received chemotherapy was 61.20%. The median follow-up period was 4 (interquartile range [IQR]: 3–10) months in the whole population, 4 (IQR: 2–10) months in the training cohort, and 5 (IQR: 2–10) months in the validation cohort. Patient clinical data are presented in **Table 1**. A P-value of less than 0.05 for the chi-square test indicated no distributional differences between the training and validation cohorts.

**Table 1:** Clinical information on patients aged ≥50 years with advanced pancreatic ductal adenocarcinoma.

Variable	Whole population		Training population		Validation population		P	Nanchong Central Hospital
	Number	%	Number	%	Number	%		149
	17,621		12,333		5,288			
Age								
50-65	6,262	35.54%	4,400	35.68%	1,862	35.21%	0.83	23
65-80	8,585	48.72%	5,983	48.51%	2,602	49.21%		84
≥80	2,774	15.74%	1,950	15.81%	824	15.58%		42
Race								
Black	2,072	11.76%	1,472	11.94%	600	11.35%	0.91	0
White	14,135	80.22%	9,861	79.96%	4,274	80.82%		0
Other	1,414	8.02%	1,000	8.11%	414	7.83%		149
Sex								
F	8,437	47.88%	5,919	47.99%	2,518	47.62%	0.56	64
M	9,184	52.12%	6,414	52.01%	2,770	52.38%		85
AJCC Stages								
III	3,087	17.52%	2,172	17.61%	915	17.30%	0.47	43
IV	14,534	82.48%	10,161	82.39%	4,373	82.70%		106
Site								
Head	8,045	45.66%	5,632	45.67%	2,413	45.63%		59
Body	3,542	20.10%	2,500	20.27%	1,042	19.70%	0.28	18
Tail	3,605	20.46%	2,512	20.37%	1,093	20.67%		32
Others	2,429	13.78%	1,689	13.69%	740	13.99%		40
Grade ^a^								
Well	1,606	9.11%	1,136	9.21%	470	8.89%	0.15	17
Bad	1,805	10.24%	1,286	10.43%	519	9.81%		23
Unknow	14,210	80.64%	9,911	80.36%	4,299	81.30%		109
CA19-9								
Positive	5,371	30.48%	3,734	30.28%	1,637	30.96%	0.63	53
Negative	12,250	69.52%	8,599	69.72%	3,651	69.04%		96
Number							0.38	
1	14,333	81.34%	10,043	81.43%	4,290	81.13%		105
>1	3,288	18.66%	2,290	18.57%	998	18.87%		44
Radiation							0.29	
Yes	1,789	10.15%	1,285	10.42%	504	9.53%		34
No	15,832	89.85%	11,048	89.58%	4,784	90.47%		115
Chemotherapy								
Yes	10,784	61.20%	7,493	60.76%	3,291	62.24%	0.36	97
No	6,837	38.80%	4,840	39.24%	1,997	37.76%		52

a Well: Grades I and II; Bad: Grades III and IV.

### Establishment of the nomogram

**Figure 2:**
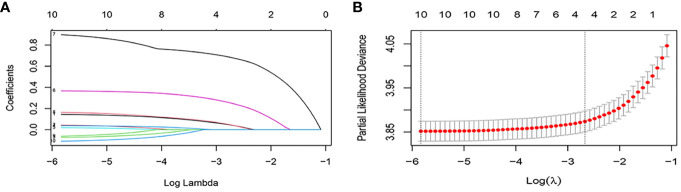
Feature selection using the LASSO Cox regression. **(A)** Profiles of lasso coefficient. **(B)** Selection of tuning parameter (lambda) in the LASSO regression using five-fold cross validation.

**Figure 3:**
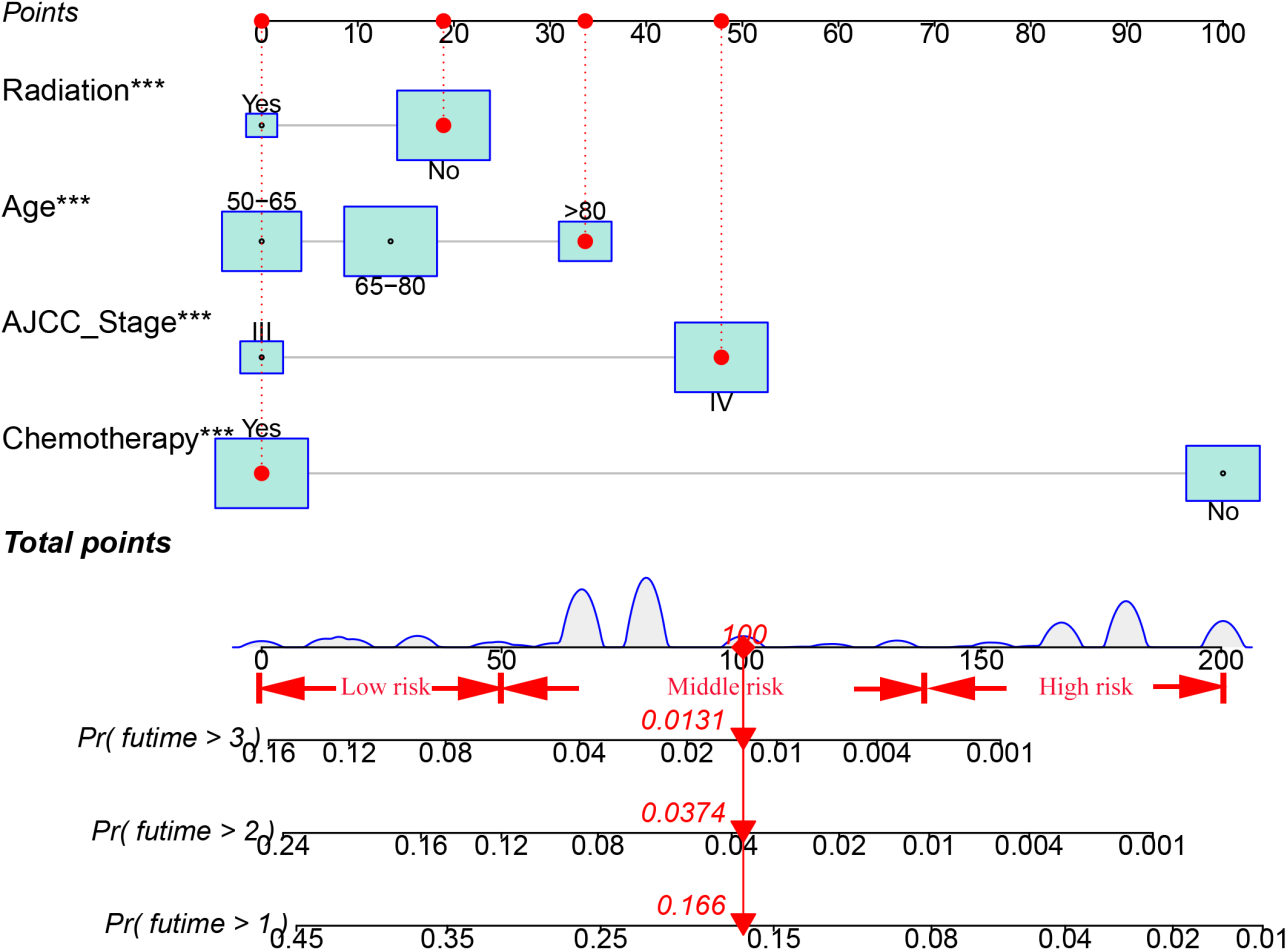
A nomogram for patients with advanced pancreatic ductal adenocarcinoma aged ≥50 years.

Ten variables were subjected to LASSO Cox regression, and four variables with non-zero coefficients were identified as significant predictors of CSS in advanced pancreatic ductal adenocarcinoma, including age, chemotherapy, radiotherapy, and AJCC stage (**Figure 2**) (**Table 2**). Therefore, all these variables were included in the new model. To utilize the new model to forecast the probability of CSS in patients with advanced pancreatic ductal adenocarcinoma, a risk score for each variable was first derived from the patient’s clinical information. Then, the sum of the scores for all variables was calculated, the location of the patient was found on the total score, and a plumb line was created through that point. The intersection of the plumb line and the three lines indicated the probability of CSS at 1, 2, and 3 years (**Figure 3**).

**Table 2:** The results of non-zero coefficients.

Variable	Coefficients
Age	0.038
AJCC Stage	0.243
Chemotherapy	0.675
Radiation	0.038

### Validation model

**Figure 4:**
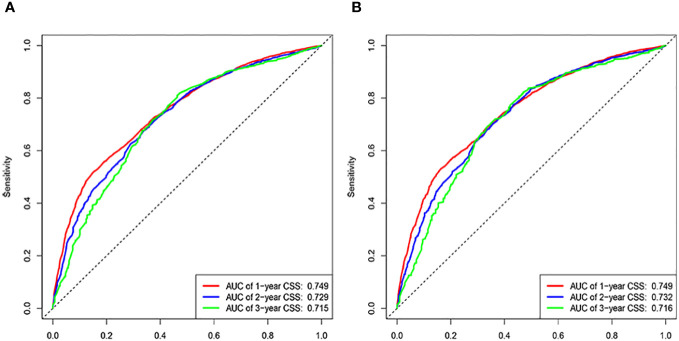
ROC curves of 1, 2, and 3 years. **(A)** Training cohorts. **(B)** Validation cohorts.

**Figure 5:**
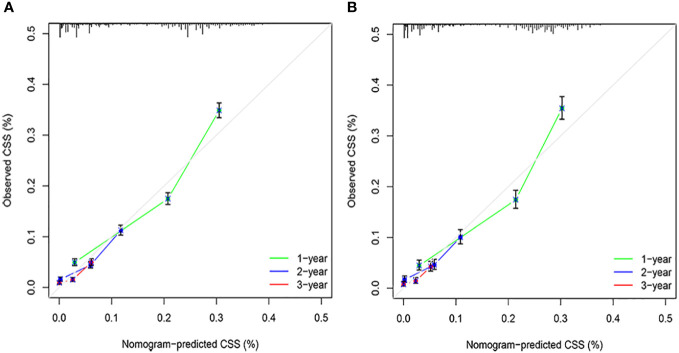
Calibration plots. **(A)** Training cohorts. **(B)** Validation cohorts.

**Figure 6:**
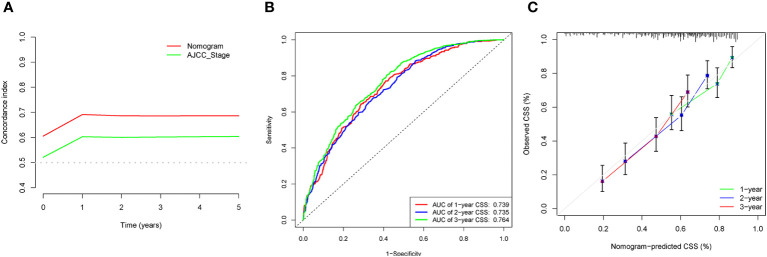
External validation of data analysis results. **(A)** C-index analysis. **(B)** Analysis of ROC curves. **(C)** Calibration curves analysis.

The C-indices associated with the nomogram were 0.721 (95% CI: 0.715–0.735) and 0.729 (95% CI: 0.719–0.738) for the training and validation cohorts, respectively. The areas under the ROC curves for the training cohort at 1, 2, and 3 years were 0.749, 0.729, and 0.715, respectively. The areas under the ROC curves for the validation cohort at 1, 2, and 3 years were 0.749, 0.732, and 0.716, respectively (**Figure 4**). The calibration curves indicated that the predicted CSS probabilities and actual CSS probabilities for the nomogram were generally consistent (**Figure 5**). The results of the external validation showed that the model not only possessed outstanding predictive ability but also excellent stability (**Figure 6**).

### Comparison of the new model and AJCC staging

**Figure 7:**
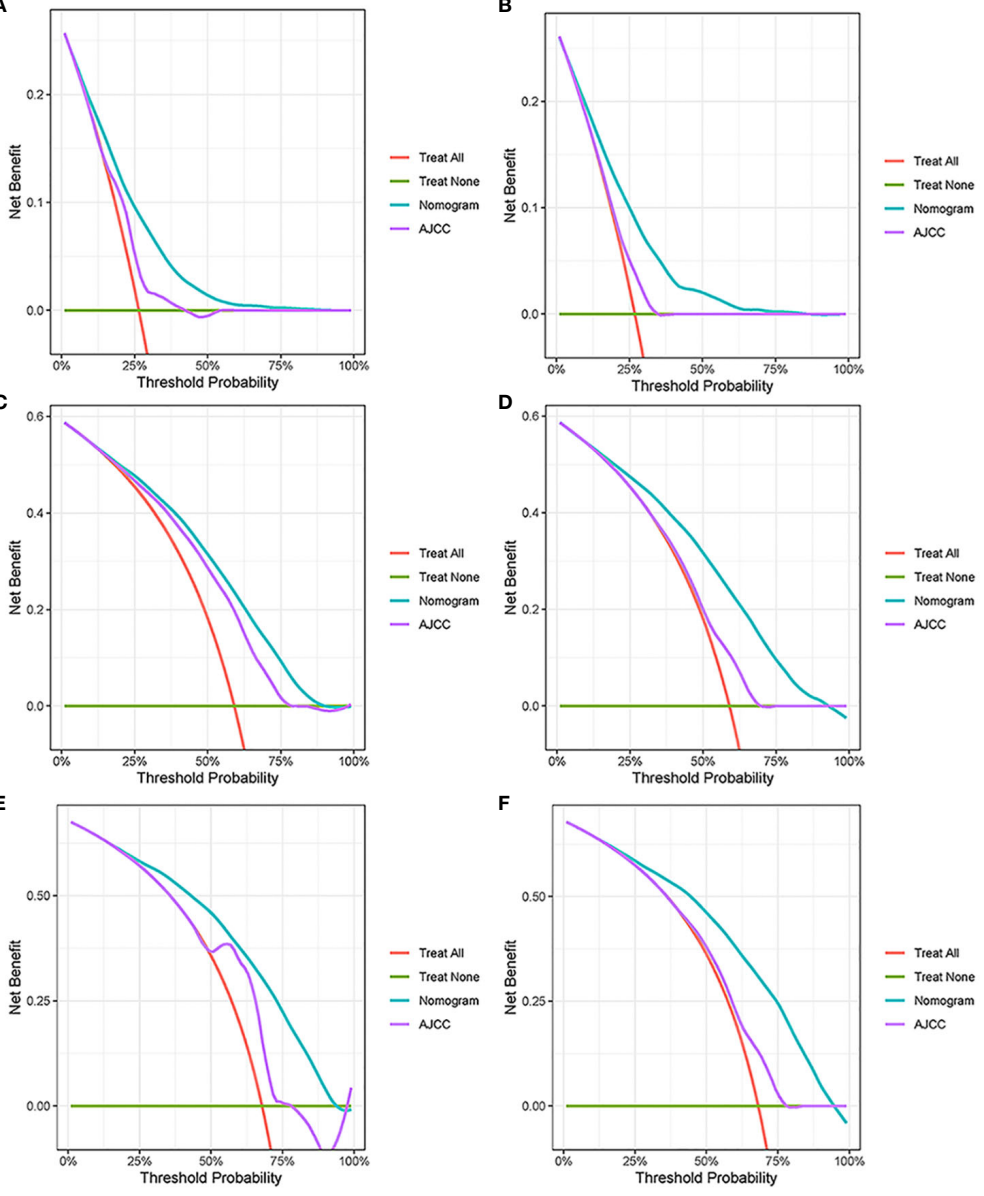
Decision curve analysis. **(A, C, E)** DCA curves in the training cohorts; **(B, D, F)** DCA curves in the validation cohorts.

**Figure 8:**
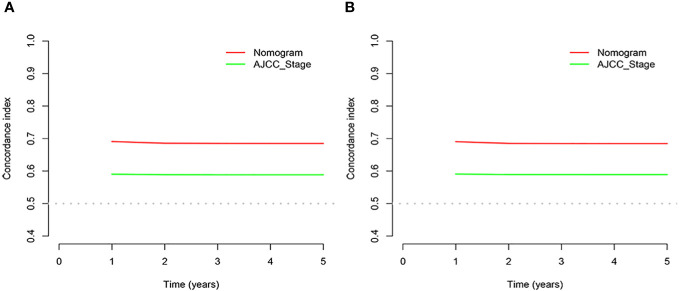
C-index plots. **(A)** Training cohorts. **(B)** Validation cohorts.

In the results of the analysis, the C-index of the nomogram for both the training and validation cohorts was higher than the AJCC staging (**Figure 7**). The 1-, 2-, and 3-year NRIs for the training cohort were 0.88 (95% CI = 0.81–0.95), 0.94 (95% CI = 0.85–0.98), and 0.72 (0.60–0.86). Meanwhile, 0.91 (95% CI = 0.79–0.97), 0.95 (95% CI = 0.82–1.13), and 0.77 (0.51–1.02) were NRIs for the validation cohorts. The 1-, 2-, and 3-year IDIs values for the training were 0.24 (95% CI = 0.22–0.26), 0.23 (95% CI = 0.18–0.28), and 0.22 (95% CI = 0.16–0.29) (P <0.001). The IDI values were 0.24 (95% CI = 0.20–0.27), 0.46 (95% CI = 0.41–0.55), and 0.27 (95% CI = 0.20–0.34) (P <0.001) for the validation cohort (**Table 3**). The DCA curves implied that the clinical benefit of the nomogram was greater than that of AJCC staging in both cohorts (**Figure 8**).

**Table 3:** IDI and NRI analysis results.

Index		Training		*P* value	Validation		*P*
		Value	95%CI		Value	95%CI	
**NRI**	1 year	0.88	0.81–0.95	–	0.91	0.79–0.97	–
	2 years	0.94	0.85–0.98	–	0.95	0.82–1.13	–
	3 years	0.72	0.60–0.86	–	0.77	0.51–1.02	–
**IDI**	1 year	0.24	0.22–0.26	<0.05	0.24	0.20–0.27	<0.05
	2 years	0.23	0.18–0.28	<0.05	0.46	0.41–0.55	<0.05
	3 years	0.22	0.16–0.29	<0.05	0.27	0.20–0.34	<0.05

### Prognostic differences between the new classification system and AJCC staging

**Figure 9:**
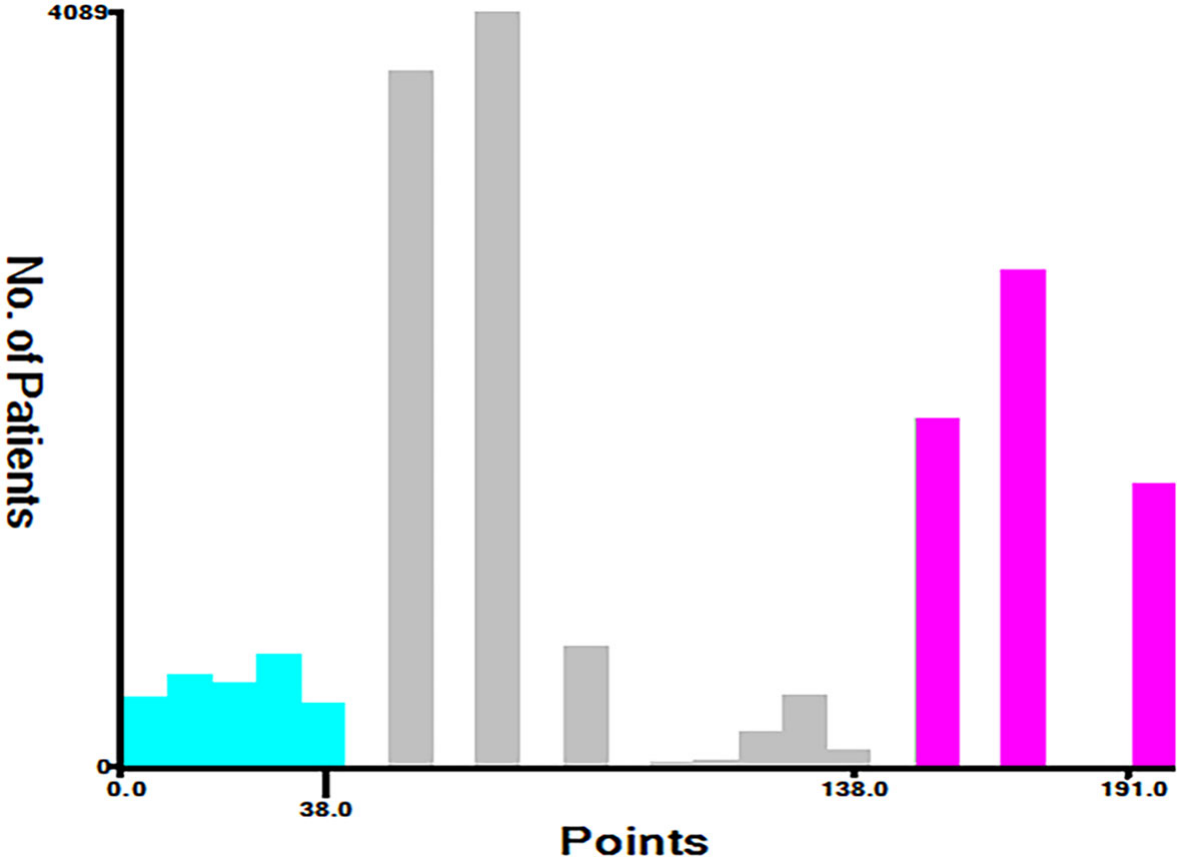
Cutoff point for risk stratifications selected using X-tile.

**Figure 10:**
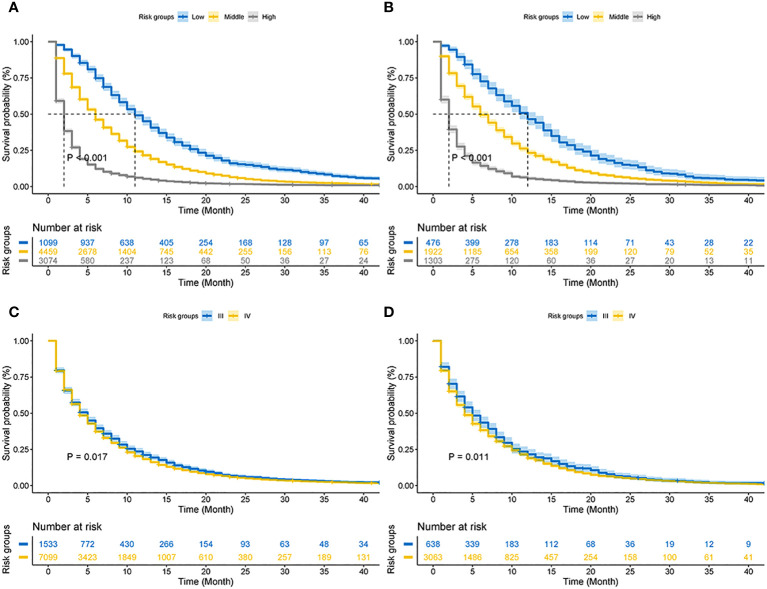
Kaplan–Meier CSS curves of advanced pancreatic ductal adenocarcinoma aged ≥50 years. **(A)** The new risk stratification system in the training cohorts. **(B)** The new risk stratification system in the validation cohorts. **(C)** AJCC staging in the training cohort. **(D)** AJCC staging in the validation cohort.

Based on the total score, patients with advanced pancreatic ductal adenocarcinoma aged ≥50 years were divided into three risk groups, low-risk (total points<50), medium-risk (50 ≤ total points <138) and high-risk (total points ≥138) (**Figure 9**) (**Supplementary 1**). The Kaplan–Meier survival curves demonstrated that the newly established classification system possesses excellent competence to differentiate patients at different risk levels compared with AJCC staging. This finding was validated in the validation cohort (**Figure 10**).

## Discussion

Ductal adenocarcinoma of the pancreas has a five-year survival rate of less than 10% and is the second leading cause of cancer-related deaths worldwide by 2030 (1, 19–21). Ductal adenocarcinoma is the leading pathological type of pancreatic malignancy (22). Due to a lack of early screening and diagnostic techniques, 90% of patients are lost to surgery at diagnosis (4, 5, 23, 24). AJCC staging is widely adopted for treatment and survival prediction of most neoplasms. However, in addition to tumor stage, a variety of factors, such as age and chemotherapy, are also factors that significantly influence CSS. Therefore, new models are required to improve the accuracy of prognosis of patients with advanced pancreatic cancer. Various studies have shown that a nomogram incorporating more variables can guide the individual prediction of survival to help clinical patients (25, 26). This study applied information from the SEER database of 17,621 patients with advanced ductal adenocarcinoma aged ≥50 years to develop a new nomogram and risk stratification system to improve the accuracy of CSS prediction for patients with advanced ductal adenocarcinoma aged ≥50 years.

Although previous studies have reported prognostic models related to pancreatic cancer, they are quite different from the present study in terms of population and methodology (27, 28). A model with a large cohort would enhance its stability and credibility. However, the small number of cases in the existing studies on pancreatic cancer and the lack of scientific validation methods in some studies certainly reduce the credibility of the results (29, 30). Age has been shown to affect the incidence and prognosis of pancreatic cancer. The role of age in advanced pancreatic cancer has further increased in severity (31, 32). Therefore, studies on the prognosis of elderly patients with advanced pancreatic cancer are crucial. However, only a few studies have focused on this topic. This study selected 10 clinical data points from patients with advanced ductal adenocarcinoma aged ≥50 years from the SEER database. LASSO Cox regression analysis showed that four clinical variables, including age, AJCC stage, radiotherapy, and chemotherapy, were the preferred combination to construct a prognostic model for patients with advanced ductal adenocarcinoma aged ≥50 years. The incidence of pancreatic ductal adenocarcinoma is mostly in older patients, with the incidence of pancreatic ductal adenocarcinoma before the age of 50 years being less than 10% (33, 34). Studies have shown that the incidence of pancreatic ductal adenocarcinoma in whites increases by approximately 6% after the age of 50 years (35, 36). The increasing proportion of the global elderly population is increasing the disease burden of pancreatic ductal adenocarcinoma (24). Klein et al. (37) discovered that the incidence of pancreatic ductal adenocarcinoma will double in the next 30 years. Global aging is an irreversible trend, and early preventive measures for pancreatic ductal carcinoma are urgently required. Advanced ductal adenocarcinoma of the pancreas was lost during the surgery. Chemotherapy and radiotherapy are the mainstay of treatment for patients with advanced pancreatic ductal adenocarcinoma. However, the clinical management of advanced pancreatic ductal adenocarcinoma remains controversial. European Society for Medical Oncology (ESMO) guidelines recommend gemcitabine-based monotherapy and capecitabine-based radiotherapy as alternative options (38). The NCCN (National Comprehensive Cancer Network) recommends that patients with advanced pancreatic ductal adenocarcinoma should receive a combination of folic acid and albumin paclitaxel + gemcitabine for 4–6 months, followed by radiotherapy (39). While the benefit of radiotherapy in patients with advanced pancreatic ductal adenocarcinoma is unclear, both ESMO and NCCN highlight the necessity of radiotherapy combined with chemotherapy in the treatment process (40, 41). Primary treatment of some patients with advanced pancreatic ductal adenocarcinoma has been successful in reducing the neoplasm size and achieving the criteria for surgery-induction chemotherapy. Considering the treatment guidelines and differences in prognosis for patients with advanced pancreatic ductal adenocarcinoma, induction chemotherapy may be used as a management approach for some patients with advanced pancreatic ductal adenocarcinoma (42–44).

Age, AJCC stage, radiotherapy, and chemotherapy were included in the line plot by analyzing 10 clinical variables. The C-index were higher than 0.7 in both the training and validation cohorts, indicating the excellent application capabilities of the nomogram. The areas under the ROC curve were 0.749, 0.729, and 0.715 for the 1, 2, and 3 years in training cohorts, respectively. The area under the ROC curve was also greater than 0.7 in the validation cohorts, indicating that the nomogram had good predictive power. The predicted and actual CSS values largely overlapped between the two cohorts. The results of the NRIs, IDIs, and C-index associated with the nomogram showed that the nomogram had stable and excellent predictive ability compared to pure AJCC standard staging. DCA curves also showed excellent clinical benefits with the nomogram. In the nomogram, each variable value was a corresponding risk score, and the total score of the patient’s risk score was calculated based on the nomogram. The X-tile software calculated the cutoff values for the risk groupings. Patients with advanced ductal adenocarcinoma aged ≥50 years were divided into a low-risk (points: 0–38), a medium-risk (points: 50–138), and a high-risk (points: 150–191) groups. KM survival curves suggested that the prognosis of patients with the new risk stratification system differed more significantly than those with AJCC staging. These results suggest that the new risk stratification system has a greater ability to identify patients with different risk factors than AJCC staging, providing a valuable instrument for the clinical treatment of patients with advanced ductal adenocarcinoma aged ≥50 years.

Although the model has strong practical applications, this study still has shortcomings. BMI and diabetes are an important factors in the prognosis of pancreatic cancer; however, there is no record of this in the SEER database. The SEER database contains mostly patients from the Americas, and clinical data from European and Asian patients are needed to further validate the model results. Finally, the absence of patient-specific treatment options recorded in the SEER database limits the practical application of the model and risk classification system.

## Conclusion

In conclusion, a prognostic nomogram for advanced pancreatic ductal adenocarcinoma aged ≥50 years was constructed using variables screened by LASSO regression. The new stratification system based on the nomogram possessed a stronger power to recognize patients with different risk groups than AJCC staging, which would give clinical decision-making an applicable tool.

## Data availability statement

The original contributions presented in the study are included in the article/**Supplementary Material**. Further inquiries can be directed to the corresponding author.

## Ethics statement

Ethical approval was not required for the study involving humans in accordance with the local legislation and institutional requirements. Written informed consent to participate in this study was not required from the participants or the participants’ legal guardians/next of kin in accordance with the national legislation and the institutional requirements.

## Author contributions

YP: Writing – review & editing. YF: Formal Analysis, Writing – original draft. JY: Conceptualization, Writing – original draft. WD: Data curation, Writing – original draft. YC: Formal Analysis, Writing – review & editing. XL: Data curation, Writing – review & editing.
