# Corrigendum: LASSO-derived prognostic model predicts cancer-specific survival in advanced pancreatic ductal adenocarcinoma over 50 years of age: a retrospective study of SEER database research

**DOI:** 10.3389/fonc.2024.1370998

**Published:** 2024-02-07

**Authors:** Yuan Feng, Junjun Yang, Wentao Duan, Yu Cai, Xiaohong Liu, Yong Peng

**Affiliations:** Department of Hepatobiliary Pancreatic and Spleen Surgery, Nanchong Central Hospital, The Second Clinical Medical College, North Sichuan Medical College, Nanchong, China

**Keywords:** advanced pancreatic ductal adenocarcinoma, nomogram, AJCC staging, risk stratification, cancer-specific survival

## Text correction

In the published article, there was an error. “Nanchang” is the wrong spelling. The correct spelling should be “Nanchong.”

This correction has been applied to the Abstract, the Methodology under the External Validation section, and to the first line of **Table 1**.

**Table 1:** Clinical information on patients aged ≥50 years with advanced pancreatic ductal adenocarcinoma.

Variable	Whole population		Training population		Validation population		P	Nanchong Central Hospital
	Number	%	Number	%	Number	%		149
	17,621		12,333		5,288			
Age								
50-65	6,262	35.54%	4,400	35.68%	1,862	35.21%	0.83	23
65-80	8,585	48.72%	5,983	48.51%	2,602	49.21%		84
80	2,774	15.74%	1,950	15.81%	824	15.58%		42
Race								
Black	2,072	11.76%	1,472	11.94%	600	11.35%	0.91	0
White	14,135	80.22%	9,861	79.96%	4,274	80.82%		0
Other	1,414	8.02%	1,000	8.11%	414	7.83%		149
Sex								
F	8,437	47.88%	5,919	47.99%	2,518	47.62%	0.56	64
M	9,184	52.12%	6,414	52.01%	2,770	52.38%		85
AJCC Stages								
III	3,087	17.52%	2,172	17.61%	915	17.30%	0.47	43
IV	14,534	82.48%	10,161	82.39%	4,373	82.70%		106
Site								
Head	8,045	45.66%	5,632	45.67%	2,413	45.63%		59
Body	3,542	20.10%	2,500	20.27%	1,042	19.70%	0.28	18
Tail	3,605	20.46%	2,512	20.37%	1,093	20.67%		32
Others	2,429	13.78%	1,689	13.69%	740	13.99%		40
Grade ^a^								
Well	1,606	9.11%	1,136	9.21%	470	8.89%	0.15	17
Bad	1,805	10.24%	1,286	10.43%	519	9.81%		23
Unknow	14,210	80.64%	9,911	80.36%	4,299	81.30%		109
CA19-9								
Positive	5,371	30.48%	3,734	30.28%	1,637	30,96%	0.63	53
Negative	12,250	69.52%	8,599	69.72%	3,651	69.04%		96
Number							0.38	
1	14,333	81.34%	10,043	81.43%	4,290	81.13%		105
>1	3,288	18.66%	2,290	18.57%	998	18.87%		44
Radiation							0.29	
Yes	1,789	10.15%	1,285	10.42%	504	9.53%		34
No	15,832	89.85%	11,048	89.58%	4,784	90.47%		115
Chemotherapy								
Yes	10,784	61.20%	7,493	60.76%	3,291	62.24%	0.36	97
No	6,837	38.80%	4,840	39.24%	1,997	37.76%		52

a Well: Grades I and II; Bad: Grades III and IV.

The authors apologize for this error and state that this does not change the scientific conclusions of the article in any way. The original article has been updated.

