# Supplementary figures and images for: LASSO-derived prognostic model predicts cancer-specific survival in advanced pancreatic ductal adenocarcinoma over 50 years of age: a retrospective study of SEER database research
Source: Front Oncol. 2024 Jan 15;13:1336251. doi: 10.3389/fonc.2023.1336251 (PMC10822877; doi:10.3389/fonc.2023.1336251)

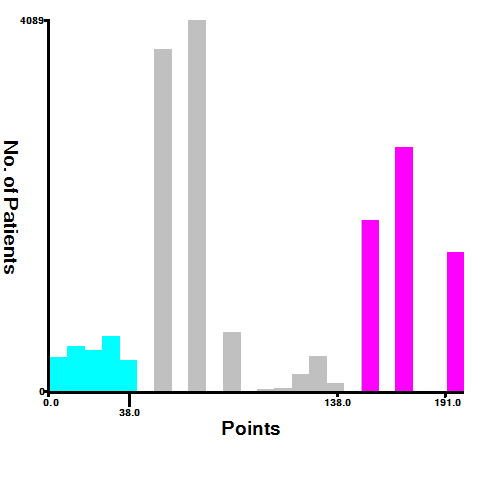

Supplement: Supplementary file 1 [file Image_1.tif]
